# Pharmacological Inhibition of Class IIA HDACs by LMK-235 in Pancreatic Neuroendocrine Tumor Cells

**DOI:** 10.3390/ijms19103128

**Published:** 2018-10-12

**Authors:** Julia Wanek, Martin Gaisberger, Marlena Beyreis, Christian Mayr, Katharina Helm, Florian Primavesi, Tarkan Jäger, Pietro Di Fazio, Martin Jakab, Andrej Wagner, Daniel Neureiter, Tobias Kiesslich

**Affiliations:** 1 Department of Internal Medicine I, Paracelsus Medical University/Salzburger Landeskliniken (SALK), 5020 Salzburg, Austria; julia.wanek@sbg.ac.at (J.W.); christian.mayr@pmu.ac.at (C.M.); and.wagner@salk.at (A.W.); 2 Biomedical Sciences, Salzburg University of Applied Sciences, 5412 Puch/Salzburg, Austria; 3 Department of Biosciences, Research Division of Regeneration, Stem Cell Biology and Gerontology, University of Salzburg, 5020 Salzburg, Austria; 4 Institute of Physiology and Pathophysiology, Paracelsus Medical University, 5020 Salzburg, Austria; martin.gaisberger@pmu.ac.at (M.G.); marlena.beyreis@pmu.ac.at (M.B.); martin.jakab@pmu.ac.at (M.J.); 5 Department for Radon Therapy Research, Ludwig Boltzmann Institute for Arthritis and Rehabilitation, Institute of Physiology and Pathophysiology, Paracelsus Medical University, 5020 Salzburg, Austria; 6 Gastein Research Institute, Institute of Physiology and Pathophysiology, Paracelsus Medical University, 5020 Salzburg, Austria; 7 Institute of Pathology, Paracelsus Medical University/Salzburger Landeskliniken (SALK), 5020 Salzburg, Austria; k.helm@salk.at (K.H.); d.neureiter@salk.at (D.N.); 8 Cancer Cluster Salzburg, 5020 Salzburg, Austria; 9 Department of Visceral, Transplant and Thoracic Surgery, Medical University of Innsbruck, 6020 Innsbruck, Austria; florian.primavesi@tirol-kliniken.at; 10 Department of Surgery, Paracelsus Medical University/Salzburger Landeskliniken (SALK), 5020 Salzburg, Austria; ta.jaeger@salk.at; 11 Department of Visceral Thoracic and Vascular Surgery, Philipps University Marburg, 35033 Marburg, Germany; difazio@med.uni-marburg.de

**Keywords:** pancreatic neuroendocrine tumors, histone deacetylases, LMK-235, cytotoxicity, apoptosis

## Abstract

Histone deacetylases (HDACs) play a key role in epigenetic mechanisms in health and disease and their dysfunction is implied in several cancer entities. Analysis of expression patterns in pancreatic neuroendocrine tumors (pNETs) indicated HDAC5 to be a potential target for future therapies. As a first step towards a possible treatment, the aim of this study was to evaluate the in vitro cellular and molecular effects of HDAC5 inhibition in pNET cells. Two pNET cell lines, BON-1 and QGP-1, were incubated with different concentrations of the selective class IIA HDAC inhibitor, LMK-235. Effects on cell viability were determined using the resazurin-assay, the caspase-assay, and Annexin-V staining. Western Blot and immunofluorescence microscopy were performed to assess the effects on HDAC5 functionality. LMK-235 lowered overall cell viability by inducing apoptosis in a dose- and time-dependent manner. Furthermore, acetylation of histone-H3 increased with higher LMK-235 concentrations, indicating functional inhibition of HDAC4/5. Immunocytochemical analysis showed that proliferative activity (phosphohistone H3 and Ki-67) decreased at highest concentrations of LMK-235 while chromogranin and somatostatin receptor 2 (SSTR2) expression increased in a dose-dependent manner. HDAC5 expression was found to be largely unaffected by LMK-235. These findings indicate LMK-235 to be a potential therapeutic approach for the development of an effective and selective pNET treatment.

## 1. Introduction

Pancreatic neuroendocrine tumors (pNETs) are a heterogeneous group of malignancies originating from cells of the diffuse endocrine system of the pancreas [1]. Only 1–5% of patients with pancreatic tumors are diagnosed with pNETs, resulting in an overall incidence lower than 1/100,000 cases per year [2,3]. Functional pNETs display distinct symptoms due to hypersecretion of specific hormones, including insulin (insulinoma), gastrin (gastrinoma), glucagon (glucagonoma), and vasoactive intestinal polypeptide (VIPoma), whereas non-functional pNETs display unspecific symptoms or stay asymptomatic [1,4,5]. Compared to other pancreatic tumors, pNETs are associated with a better prognosis, but still lack specific treatment options [6,7]. Multimodal therapy with surgical removal of the primary tumor and regional lymph nodes combined with chemotherapeutic drugs represents the currently established treatment [5,6]. Thus far, many underlying mechanisms of pNET tumorigenesis and tumor progression as well as risk factors are still unknown. Recently, alterations of epigenetic mechanisms have been found in several malignancies [8,9,10]. Histone deacetylases (HDACs) play a key role in epigenetics by altering post-translational histone modifications through removal of the acetyl group of N-terminal lysine histone residues [11], thus repressing expression through tighter DNA-packaging around histones. Dysfunction and or expression changes of HDACs have been observed in a wide range of tumors, including breast cancer, melanoma, and prostate cancer [10,12,13,14,15,16]. This led to the development of the first pharmaceutical HDAC inhibitor (HDACi), which received FDA approval in 2006, and several more being recently approved or being investigated in trials [17,18,19]. A recent report describes a phase II study on the use of the pan-HDAC inhibitor, panobinostat, in 15 low-grade NET patients indicating a low response rate, but encouraging stable disease rate and reasonable progression-free survival [20]. In 2017, a retrospective immunohistochemical study of resected pNET tissues evaluated changes in HDACs expression compared to normal tissue [21]. Here, poor clinical outcome, i.e., low overall and disease-free survival, was associated with overexpression of HDAC5 in pNET tissue [21]. Additionally, expression changes of HDAC5 were found, e.g., in hepatocellular carcinoma [22,23], lung cancer [24], and colon cancer [25], thus underlining its role in oncogenesis. Several studies, additionally, have shown a significant cytotoxic effect of various HDAC inhibitors (mostly pan-HDAC specificity) in pNET cell lines in vitro [26,27,28,29,30]. Therefore, in the current study, we analyzed the in vitro effects of pharmacological HDAC inhibition (LMK-235, a selective class IIA HDAC inhibitor [31]) in two established pNET cell lines, BON-1 and QGP-1, to determine whether LMK-235 represents an effective modifier of this epigenetic mechanism and a possible therapeutic approach in pNET.

## 2. Results

### 2.1. Cell Viability

**Figure 1:**
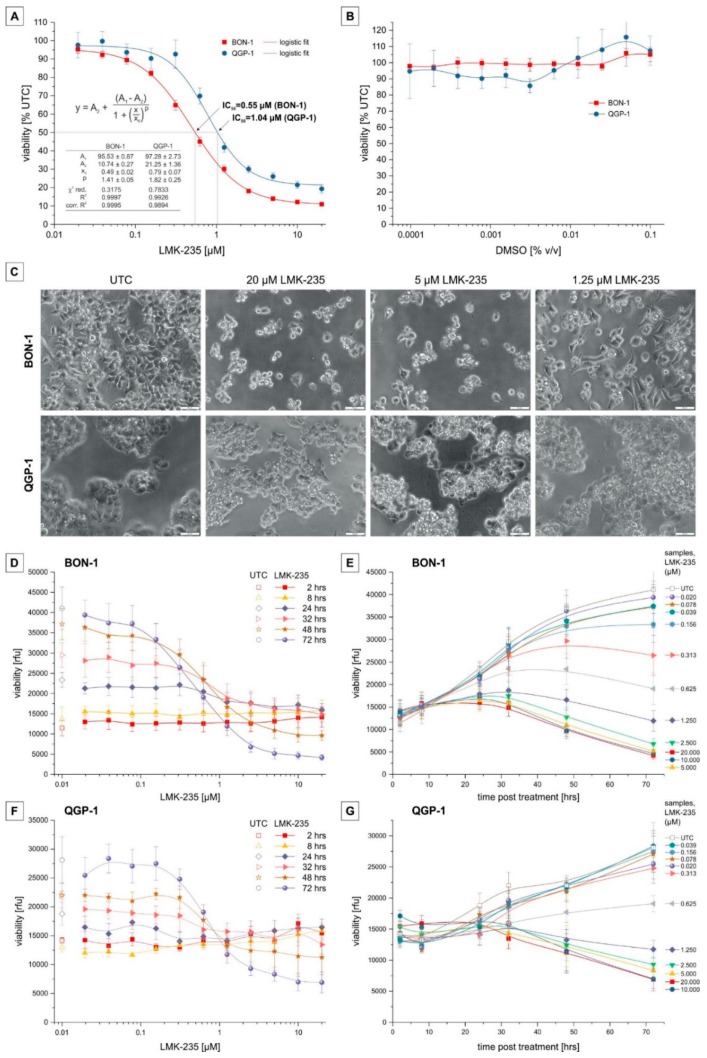
Cytotoxicity of LMK-235 on BON-1 and QGP-1. BON-1 and QGP-1 were incubated with different LMK-235 concentrations for up to 72 h. Viability was measured by the resazurin assay and displayed either as the percentage of untreated control (UTC) or relative fluorescence units (rfu). (**A**,**B**) 72 h treatment with a serial dilution of LMK-235 between 0.02–20 µM (**A**) for BON-1 (red; *n* = 9) and QGP-1 (blue; *n* = 8) cells and corresponding DMSO concentrations (**B**; *n* = 3). Data points represent mean ± SEM, fitted based on a logistic fit function (**A**). (**C**) Phase contrast images (200× magnification) of BON-1 and QGP-1 treated for 72 h with 20, 5, and 1.25 µM LMK-235. Scale bar indicates 50 µm. (**D**,**F**) Cell viability displayed as absolute fluorescence units for BON-1 (**D**) and QGP-1 (**F**) incubated for different periods (2, 8, 24, 32, 48, 72 h) with LMK-235 concentrations ranging from 0.02 to 20 µM. (**E**,**G**) Cell viability displayed as absolute fluorescence units for BON-1 (**E**) and QGP-1 (**G**) treated with different LMK-235 concentrations (0.02–20 µM) for 2, 8, 24, 32, 48, or 72 h. (**D**–**G**) Data points represent means ± SEM of three experiments, interpolated with a B-spline function. Abbreviations: UTC = untreated control, rfu = relative fluorescence units.

To determine the effects of LMK-235 on cell viability, we performed a resazurin assay after incubation with different LMK-235 concentrations as well as untreated control cells over a time course of up to 72 h (Figure 1A). Solvent control was performed with the corresponding DMSO (dimethyl sulfoxide) concentrations for 72 h incubation and showed no effect on cell viability in both cell lines (Figure 1B).

Treatment with LMK-235 showed a dose-dependent decrease in viability in both cell lines after a 72 h incubation period (Figure 1A). Based on a logistic fit, IC_50_ values are 0.55 µM (95% CI 0.52–0.58 µM) and 1.04 µM (95% CI 0.89–1.18 µM) for BON-1 and QGP-1 cells, respectively. Decreased viability and morphological changes were also visible by light microscopy for both cell lines (Figure 1C): For BON-1 cells, with increasing concentrations of LMK-235, the cell number decreases and the cells become round and less adherent. In the case of QGP-1, LMK-235 causes an increase in cellular contrast and structure—observations consistent with an apoptotic phenotype for both cell lines. Results from viability time series (Figure 1D–G) revealed that incubation with 2.5, 5, 10, and 20 µM LMK-235 led to a reduction of viable cells below the initial value when incubated longer than 48 h, indicating direct cytotoxicity and cell death. BON-1 showed a continuous dose-dependent reduction of viability whereas QGP-1 showed a rather dichotomous response with cell survival at low concentrations (<0.31 µM) and a dose-dependent reduction of cell viability at concentrations >2.25 µM LMK-235.

### 2.2. Apoptosis Induction

**Figure 2:**
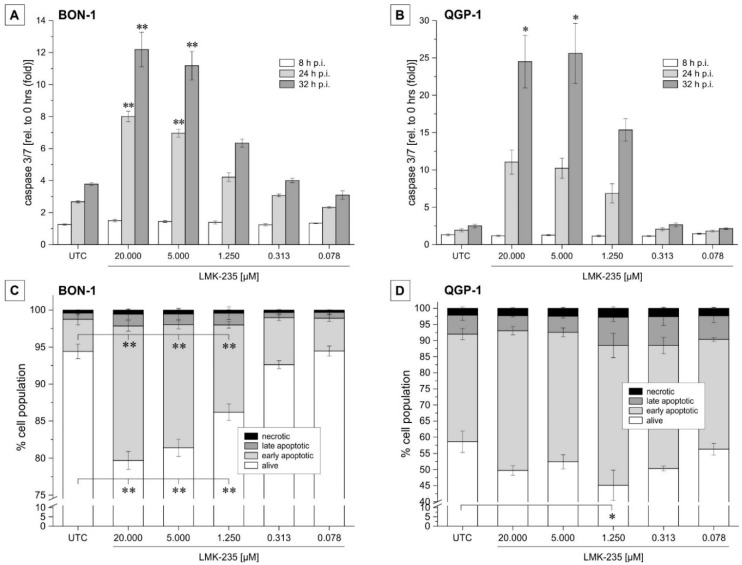
LMK-235 effects on apoptosis induction in pNET cells. (**A**,**B**) BON-1 (**A**) and QGP-1 (**B**) were incubated for 8, 24, and 32 h with different LMK-235 concentrations (0.078–20 µM). Relative changes in caspase activity were measured as a parameter for treatment-induced apoptosis. Bars represent mean ± SEM for *n* = 4 experiments. (**C**,**D**) Flow cytometry results of Annexin V/7-AAD staining are shown for BON-1 (**C**) and QGP-1 (**D**). Bars represent the cumulative percentages (*n* = 3) for alive, early, or late apoptotic and necrotic cells when treated for 24 h with LMK-235 (0.078–20 µM). Asterisks indicate *p*-values of <0.05 (one) or <0.01 (two) versus corresponding untreated control. Abbreviations: p.i. = post incubation, UTC = untreated control.

Earlier studies found that HDAC5 inhibition induces apoptosis in cancer cells [13]. Therefore, we evaluated the induction of apoptosis as a response to LMK-235 treatment by measuring caspase activity. Caspase 3/7 activity assay was performed at the time of incubation (0 h) and after 8, 24, and 32 h’ post incubation. BON-1 cells showed a highly significant (*p* < 0.01) increase in caspase activity when treated with 20 or 5 µM LMK-235 for 24 and 32 h compared to the caspase activity at the time point of incubation (Figure 2A,B). For QGP-1, a significant change was observed with 20 and 5 µM LMK-235 after 32 h’ incubation. For all other LMK-235 concentrations, a dose- and time-dependent trend was observed for both cell lines (Figure 2A,B). Control experiments performed with corresponding amounts of the solvent (DMSO) yielded caspase 3/7 activities in the range of untreated controls (data not shown).

In addition, the early apoptosis marker, Annexin-V, and late-stage apoptosis marker, Annexin-V/7-AAD, expression were determined by flow cytometry. The cell fraction exposing the early apoptosis surface marker, Annexin-V, significantly increased in BON-1 cells (*p* < 0.01) when incubated with 20, 5, and 1.25 µM LMK-235 for 24 h (Figure 2C). Changes in late-stage apoptotic cell population (Annexin-V/7-AAD) were rather small and not significant. In QGP-1 cells (Figure 2D), changes in Annexin-V surface expression were not as pronounced as in BON-1 cells and were not observable in a distinct dose-dependent manner.

### 2.3. Effect on Acetyl-H3 Expression

**Figure 3:**
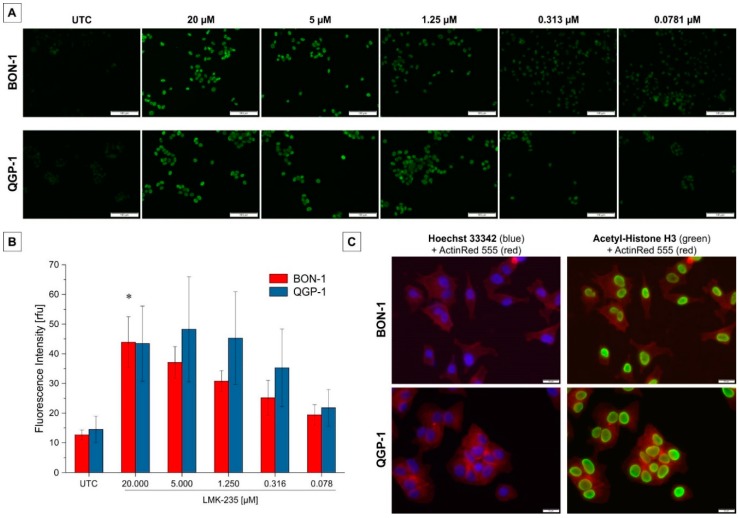
Acetyl-H3 immunofluorescence following LMK-235 treatment. (**A**) Results of one representative acetylated H3 immunofluorescence staining is shown for a LMK-235 dilution series for both cell lines. All samples were visualized using the same exposure time. Acetylated histone H3 is stained in green and the scale bar indicates 100 µm. Magnification 100×. (**B**) Quantitative immunofluorescence intensities are represented as bars for mean ± SEM. * indicate *p* < 0.05. (**C**) Overlay of ActinRed (red, cytoskeleton) with Hoechst 33342 (blue, nuclei) or acetyl-H3 (green) staining demonstrating the localization of acetyl-H3 staining exclusively in the cells’ nuclei. Magnification 200×. Abbreviations: UTC = untreated control.

Effects of LMK-235 on HDAC4/5 function was evaluated at the level of histone H3 acetylation using immunofluorescences and immunoblotting. Changes in immunofluorescence signal intensity for acetyl-histone H3 are shown qualitatively (Figure 3A) and after quantitative assessment (Figure 3B). A dose-dependent tendency was observed for both BON-1 and QGP-1 with a significant increase (*p* < 0.05) in H3 acetylation intensity for BON-1 cells incubated with 20 µM LMK-235 for 24 h. Comparison of immunofluorescence staining with ActinRed and Hoechst or anti-acetyl-H3 demonstrated that acetylated histone H3 staining is localized in the nucleus (Figure 3C).

**Figure 4:**
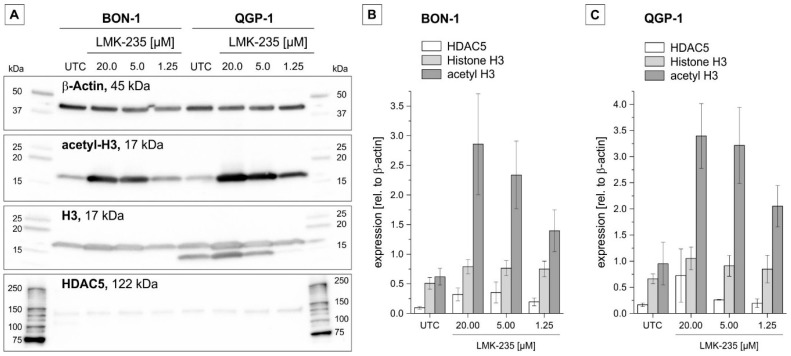
(Acetyl-) Histone H3 and histone deacetylase-5 protein expression following LMK-235 treatment. (**A**) Levels of acetyl-histone H3, histone H3, and HDAC5 were evaluated by immunoblotting after LMK-235 incubation for 24 h, as shown by one representative example blot. β-actin was used as a loading control for each sample. The β-actin expression corresponding to the HDAC5 blot is not shown. (**B**,**C**): Bars represent the semi-quantitative expression as mean values ± SEM (*n* = 3) of band intensities of BON-1 (**B**) and QGP-1 (**C**) cells. Abbreviations: UTC = untreated control.

Similar results were obtained with Western blot (Figure 4): While the expression levels of histone 3 and HDAC5 itself remain nearly constant (only a small, not significant, trend for increased expression of HDAC5), the level of acetylated histone H3 shows a clear, dose-dependent increase after LMK-235 treatment compared to untreated control cells (Figure 4).

### 2.4. Effect of LMK-235 on Expression of Selected pNET-Relevant Proteins

**Figure 5:**
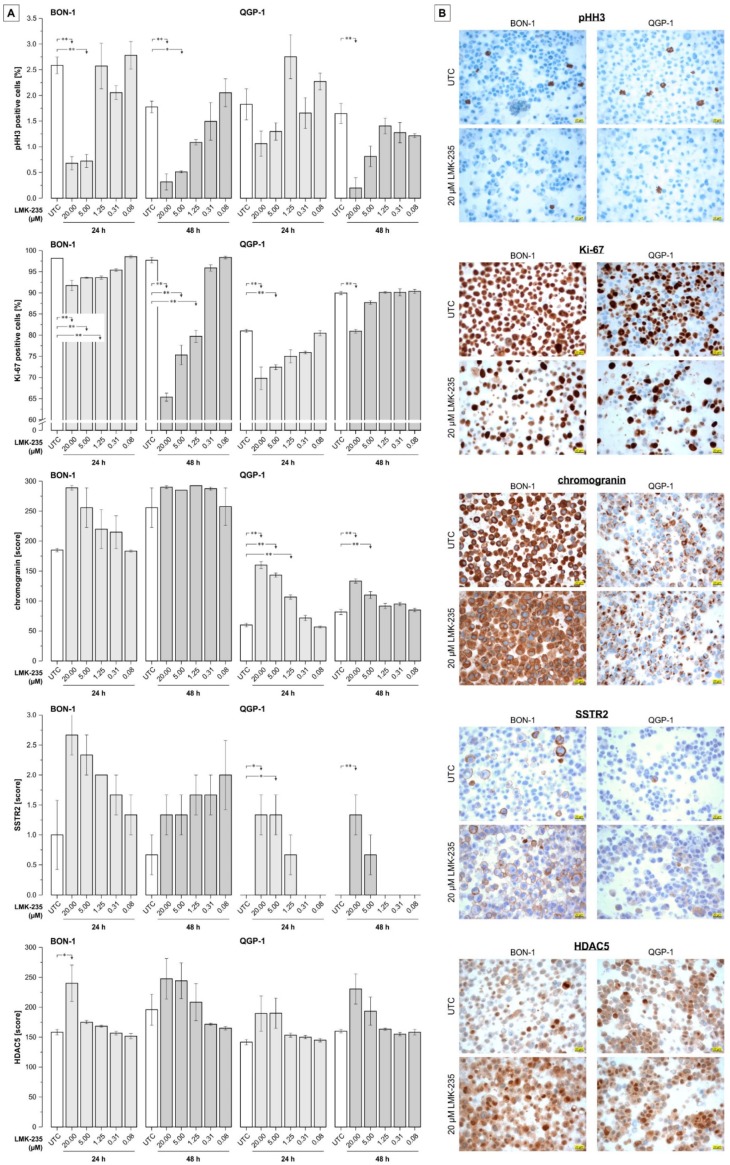
Immunocytochemistry on cell blocks of LMK-235-treated pNET cells. (**A**) Semiquantitative evaluation of immunostaining for phosphohistone H3 (pHH3) and Ki-67 (% positive cells), chromogranin (score, based on multiplication of intensity and extensity; 0–300), SSTR2 (score; 0–3), and histone deacetylase-5 (HDAC5 score; 0–300). Cells were treated with 0.08–20 µM LMK-235 for 24 or 48 h and results represent mean values ± SEM of three independent images. Asterisks indicate *p*-values of <0.05 (one) or <0.01 (two) for comparison of treatments *versus* the corresponding untreated control (statistical results for group comparisons within treatments are not shown). (**B**). Representative images of the immunostainings for UTC and LMK-235-treated (20 µM, 24 h) samples. Magnification 400× for all images, scale bars indicates 20 µm. Abbreviations: UTC = untreated control.

For further evaluation of changes in the expression of selected proteins/markers, cells were treated for 24 or 48 h, prepared as cell blocks, and stained using standard immunohistochemical techniques. As shown in Figure 5, all effects (partly significant) on protein expression compared to untreated control samples are seen for the highest concentrations of LMK-235 (1.25, 5, or 20 µM). While these concentrations of LMK-235 cause significant down-regulation of the expression of phosphohistone H3 (pHH3) and Ki-67 proliferation markers in both cell lines, the expression of chromogranin and the somatostatin receptor, SSTR2, increased dose-dependently. For QGP1 cells, SSTR2 is only detectable after incubation with 5 and 20 µM LMK-235 whereas untreated cells and those treated with concentrations <1.25 µM LMK-235 showed no SSTR2 expression. While insulin and serotonin expression could not be detected (regardless of LMK-235 treatment, data not shown), the expression of HDAC5 showed a slightly increased expression compared to UTC samples (approximately +10–20%) at the two highest concentrations of LMK-235 (5 and 20 µM).

## 3. Discussion

HDAC5 expression was investigated in several tumor entities and its oncogenic de-regulation has been found to be dependent on the cancer type [13,14]. HDAC5 over-expression has been demonstrated in human pNET tissue samples by Klieser et al. [21] and correlated with poor clinical outcome, especially in terms of low overall and disease-free survival. Similar results for HDAC5 expression were found in breast cancer tissue samples [13], hepatocellular carcinoma tissue samples [23], and melanoma cells in vitro [14]. LMK-235, a novel HDAC class IIA HDACi specifically inhibiting HDAC5 (IC_50_ = 4.22 nM) and HDAC4 (IC_50_ = 11.9 nM), was found to be cytotoxic in several human cancer cell lines [31]. Relating to the mechanisms of LMK-235’s cytotoxic action, studies performed on lung cancer cells indicate that HDAC5 inhibition with LMK-235 interferes with the ERK-1/2 pathway involved in the differentiation of cancer stem cells [32]. Recently, Li et al. identified Bcl-2-associated transcription factor 1 (BCLAF1) as a potential epigenetic therapeutic target involved in LMK-235-mediated apoptosis in diffuse large B-cell lymphoma cells [33]. Based on these results, we analyzed the cellular (cytotoxic) effects of LMK-235 treatment in an in vitro pNET model.

In the present study, we found a direct dose- and time-dependent cytotoxic effect of the HDAC5 inhibitor, LMK-235, in the pNET cell lines, BON-1 and QGP-1, resulting in a considerable loss of viability at concentrations >0.5 µM after 72 h of incubation. The IC_50_ values are in the same range as previously published results (0.49–4.62 and 0.32–2.48 µM LMK-235 for cisplatin-sensitive and -resistant cell lines, respectively [31], and ~1 µM for urothelial carcinoma cell lines [34]). Of note, BON-1 cells are more sensitive compared to QGP-1 cells (IC_50_ values of 0.55 versus 1.04 µM, respectively). To verify the underlying mechanism causing this decrease in viability, caspase activity was determined as a parameter for apoptosis. Loss in viability was accompanied by an increase in apoptosis, again in a dose- and time-dependent manner. Results for QGP-1 cells varied in a wider range due to their sensitivity to all handling steps and considerable clustering of cells. Inhibition of cell growth and induction of apoptosis by HDAC4/5 inhibition using LMK-235 has been reported for different cancer cell lines as well as chemoresistant cancer cell lines [13,23,31,35]. Still, the exact molecular mechanism inducing apoptosis remains unclear. Several previous studies have provided data on the pathophysiologic role of HDAC5 in cancer: HDAC5 was found to regulate heterochromatin assembly and cell proliferation in cancer cells [35] and to enhance the effect of chemotherapy [13,35]. Also, the stability, localization, and activity of the tumor suppressor, p53, is supported by HDAC5 inhibition in hepatocellular carcinoma cells [23]. In contrast, Huang et al. found HDAC5 over-expression to inhibit cell growth and induce apoptosis in sarcoma cells [36]. While the effect of HDAC4/5 inhibition described in the current study clearly indicates an oncogenic function of this enzyme in pNET cells, further research is necessary to elucidate the exact signaling mechanism by which LMK-235 exerts its cytotoxic function in pNET cells.

In line with previous results by Guo et al. [37], proof of functional HDAC inhibition with LMK-235 was shown with immunoblotting analyses where a dose-dependent increase of histone-H3 acetylation could be observed after treatment with LMK-235. On the contrary, no effect of LMK-235 on the overall histone-H3 and no or a slight up-regulation of HDAC5 levels were found by Western blot analysis and immunocytochemistry. The latter might be indicative of a response mechanism exerted by pNET cells to compensate for the pharmacological inhibition of HDAC5 by LMK-235, which has been predicted to bind at the catalytic site of HDAC enzymes [31]. These findings suggest that LMK-235 treatment specifically targets the enzymatic function of HDACs without dramatically altering the overall protein expression of both the enzyme and its histone target.

Immunocytochemical analyses of cell blocks showed down-regulation of proliferation markers (pHH3 and Ki-67) after treatment with LMK-235 and increased expression (in BON-1 cells) or even re-expression (in QGP-1 cells) of chromogranin and the somatostatin receptor, SSTR2 – both indications of a phenotype stabilization or higher status of cellular differentiation by LMK-235 treatment. Up-regulation or re-expression of SSTR2 is especially interesting since previous reports demonstrated a positive predictive value of SSTR2 expression on clinical outcomes of pNET patients [38,39], and somatostatin receptors and somatostatin analogs (SSAs) are currently being evaluated as potential therapeutic targets and agents, respectively [40,41]. Furthermore, for treatment with peptide receptor radionuclide therapy (PRRT), the response is highly dependent on SSTR expression as mostly quantified by pretreatment receptor-specific PET-CT [42,43].

As HDACs are known to represent important epigenetic modulators in tumorigenesis and tumor progression, several HDACis have been approved by the FDA or are in phase II and III clinical studies for the treatment of several tumor entities. Approved pan-HDACis inhibiting all HDAC classes include Vorinostat [17], Belinostat [44], and Panobinostat [19] as well as Romidepsin, a selective HDAC1 and -2 inhibitor [18]. Various other HDACis are currently in different phases of clinical trials and might achieve FDA approval within the next years [16]. Selective HDACis for specific treatment in selected and responsive tumor entities could provide an improvement for patients’ prognosis and overall treatment efficiency by limiting and significantly reducing possible off-target side effects. It is important to note that HDAC expression profiles and its function as a tumor suppressor or oncogene vary among different cancer types, thus making it hard to form generalized hypotheses and probably requiring HDAC in situ profiling in each clinical case. Therefore, specific HDACis must be tested for each malignancy for their effectiveness to assess their possible clinical efficiency.

As limitations of the present study, it should be noted that the results need to be confirmed in subsequent studies using in vivo conditions (animal models). Additionally, although the IC_50_ values of LMK-235 are roughly three times higher for HDAC4 compared to HDAC5 [31], it cannot be ruled out that in pNET cells, LMK-235 exerts it cytotoxic action also by inhibition of HDAC4.

Taken together, our study indicates LMK-235 to be a potential therapeutic approach based on HDAC inhibition and subsequent significant cytotoxicity in pNET cancer cells. LMK-235 and probably other specific HDAC5-targeting agents represent promising drug candidates for further pre-clinical testing, especially, but not limited to, pNET.

## 4. Materials and Methods

### 4.1. Cell Culture and Treatment

Cells were cultured under standard conditions at 37 °C in a humidified atmosphere containing 5% CO_2_. BON-1 cells were originally obtained from a peripancreatic lymph node from a patient with metastatic pancreatic cancer by Evers et al. [45,46]. Further characterization by Evers et al. showed expression of functional receptors for gastrin, somatostatin, serotonin, and acetylcholine as well as synthesis of serotonin, chromogranin A, neurotensin, and pancreastatin [45]. BON-1 cells were cultivated in DMEM medium (Gibco-ThermoFisher, Waltham, MA, USA) supplemented with 10% FBS (Gibco), 1% Penicillin/Streptomycin (GE Healthcare, Solingen, Germany), 1 mM sodium pyruvate (Sigma Aldrich, Vienna, Austria), and 10 mM HEPES (Sigma Aldrich). The QGP-1 cell line is a carcinoembryonic antigen (CEA) -secreting cell line (JCRB0183) derived from a pancreatic tumor and has been initially characterized by Kaku et al. [47]. QGP-1 standard medium consisted of RPMI-1460 medium (Merck, Vienna, Austria) supplemented with 10% FBS (Gibco), 1% penicillin/streptomycin (GE Healthcare), 1 mM sodium pyruvate (Sigma Aldrich), 10 mM HEPES (Sigma Aldrich), and 4 mM l-glutamine (PAA).

HDAC5 inhibition was performed using different concentrations of LMK-235 (Selleck Chemicals LLC, Munich, Germany), a selective histone deacetylase class IIA inhibitor, with stronger inhibition of HDAC5 than HDAC4 [31]. LMK-235 was dissolved in 100% dimethyl sulfoxide (DMSO) according to the manufacturer’s protocol to a stock concentration of 20 mM and stored at −20 °C. All LMK-235 concentrations were prepared imminent to use with serum-free DMEM or RPMI for BON-1 or QGP-1, respectively. BON-1 and QGP-1 cells were seeded at a density of 58,824 and 73,529 cells/cm^2^ growth area corresponding to 20,000 and 25,000 cells per well in a 96-well plate (0.34 cm²), respectively. After overnight incubation, cells were washed once with serum-free medium and incubated with LMK-235 in serum-free medium to avoid interaction of the drug with serum components. Duration and concentration of LMK-235 inhibition varied, depending on the experiment, as described in detail in the following sections. An untreated control was included in each experiment (UTC).

### 4.2. Viability

Cells were incubated in 96-well polystyrene cell culture plates (Greiner Bio-One, Kremsmünster, Austria) for 72 h or 2, 8, 24, 32, 48, and 72 h, respectively, with a 1:2 dilution series, starting at 20 µM of LMK-235 in serum-free medium. Each plate contained treated, untreated, and blank wells, each in quadruplicates. Afterwards, cells were incubated for 1 h with 0.5 mM resazurin solution in serum-free medium at 37 °C before the fluorescence intensity was measured using an Infinity M200 microplate reader (Tecan, Grödig, Austria) at an excitation wavelength of 535 nm, emission wavelength of 588 nm, and optimal gain settings. For time series from 2–72 h, supernatants of resazurin-incubated wells were collected and stored at −20 °C until completion of the time series. For fluorescence measurements, all plates were then thawed, mixed by gently shaking, and measured using the same gain settings.

### 4.3. Apoptosis Detection

Cells were treated with a 1:4 dilution series ranging from 20 µM to 0.078 µM LMK-235 in 96-well cell polystyrene cell culture plates (Greiner Bio-One). Caspase-Glo 3/7 assay (Promega, Mannheim, Germany) was performed after 0, 8, 24, and 32 h according to the manufacturer’s protocol. Supernatants including the reaction mixture were transferred for luminescence measurements into white 96-well plates (Corning, Kaiserslautern, Germany). Luminescence measurements were performed on the Infinity M200 reader with an integration time of 5 s.

After 24 h incubation with a LMK-235 1:4 dilution series (0.078–20 µM) in 6-well polystyrene cell culture plates (TPP), cells were harvested and stained according to the manufacturer’s instructions with Annexin-V and 7-AAD (both BioLegend, Koblenz, Germany) for FACS analysis of apoptosis on a Cell Lab Quanta SC flow cytometer (Beckman-Coulter, Vienna, Austria) and the Kaluza Analysis 1.3 software (Beckman-Coulter).

### 4.4. Effect on Acetyl-H3 Expression

For immunofluorescence, cells were grown in transparent 96-well polystyrene cell culture plates (Greiner-BioOne) and incubated with an 1:2 LMK-235 dilution series starting at 20 µM. After 24 h, cells were fixed in 4% formaldehyde (Polyscience, Hirschberg an der Bergstrasse, Germany) for 15 min at room temperature, blocked in blocking buffer (5% *v*/*v* normal goat serum (Cell Signaling Technology, Frankfurt am Main, Germany), 0.3% *v*/*v* Triton-X100 (Sigma-Aldrich) in PBS (Merck)) for 60 min at room temperature and stained with an anti-acetyl-histone H3 rabbit antibody (#9683, Cell Signaling Technologies) overnight according to the manufacturer’s protocol. Prior to immunofluorescence microscopy, cells were counter-stained with 1 µg/mL Hoechst 33342 (Fluka-Sigma Aldrich, Vienna, Austria) and 1:50 diluted ActinRed™ 555 ReadyProbes^®^ Reagent (Invitrogen-ThermoFisher, Waltham, MA, USA) according to the manufacturer’s protocol. Microscopic visualization was performed on the Olympus CKC53 inverted microscope equipped with the pE 300 module (Cool LED) light source, ET-EGFP (FITC/Cy2), and ET-DSRed (TRITC/Cy3) filter sets (both Chroma, Olching, Germany) as well as a U-FUW filter cube (Olympus, Vienna, Austria). Quantitative analysis of intensities was performed using the ImageJ 1.46 software [48]. In brief, nuclei were marked as positive particles by adjusting the threshold in an 8-bit picture and selected using the ROI Manager. Overlay of the selection on the original picture via the ROI Manager allowed intensity measurements only in selected nuclei.

For immunoblots, cells were grown in polystyrene cell culture dishes (SPL) and incubated with 20, 5, and 1.25 µM LMK-235 for 24 h. After harvesting with 1× trypsin-EDTA (GE Healthcare), cells were counted and cell pellets were stored at −20 °C. Gel electrophoresis of cell lysates was performed using the Mini-PROTEAN Tetra Vertical Electrophoresis Cell system with a discontinuous gel followed by blotting to a nitrocellulose membrane using the Trans-Blot^®^ Turbo™ Transfer System (all from Bio-Rad, Vienna, Austria). Membranes were blocked with blocking buffer (1× TBS-T, 5% *w*/*v* milk powder) and incubated with the primary antibodies overnight. Primary antibodies were anti-β-actin (#8457, 1:000), anti-acetyl-histone H3 (#9649, 1:1000), anti-histone-H3 (#4499, 1:2000, all from Cell Signaling Technology), and anti-HDAC5 (ab55403, 1:1000, Abcam, Cambridge, UK). For detection, membranes were washed 3 times with 1× TBS-T buffer and incubated with the secondary anti-rabbit-IgG antibody and HRP-conjugate followed by detection using the development reagent SignalFire Kit (Cell Signaling Technology) and the Molecular Imager GelDoc XR System (Bio-Rad) with the corresponding software Image Lab 5.2.1 (Bio-Rad) for documentation. Semi-quantitative analysis of band intensities was performed with the ImageJ 1.46 software [48].

### 4.5. Immunocytochemistry

Cells were grown in CellStar^®^ cell culture dishes and treated for 24 and 48 h with a 1:4 LMK-235 dilution series from 0.078–20 µM. After harvesting the cells with 1× trypsin-EDTA (GE Healthcare), cell blocks were prepared for each concentration using a 1:1 mix of citrate plasma and Thromborel S (Siemens Healthcare Diagnostics Products, Marburg, Germany). Four-μm sections were mounted on glass slides, deparaffinized with graded alcohols, pretreated with high pH (Ventana), and stained using the following primary antibodies: Anti-pHH3 (rabbit polyclonal (rp), Cat.-No. 760-4591, clone not specified, Ventana, ready-to-use (rtu)), anti-Ki67 (rabbit monoclonal (rm), Cat.-No. 790-4286, clone 30.9, Ventana, rtu), anti-HDAC5 (rp, Cat.-No. ab55403, clone not specified, Abcam, dilution 1:500), anti-Chromogranin A (mouse monoclonal (mm), Cat.-No. 760-2519, LK2H10, Ventana, rtu), anti-somatostatin receptor 2 (SSTR2, rp, Cat.-No. ab134152, UM1, Abcam, 1:200), anti-serotonin (mm, Cat.-No. M0758, 5HT-H209, DAKO, 1:50), and anti-insulin (guinea pig polyclonal, Cat.-No. CMC26550030, clone not specified, Cell Marque, rtu). All immunohistochemical stainings were performed on a Benchmark Ultra platform with the UltraView Universal DAB detection kit (both Ventana-Roche, Vienna, Austria). Due to different cellular localizations of the used antibodies (nuclear: Ki67, pHH3, and HDAC5; cytoplasmatic: HDAC5, chromogranin, serotonin, and insulin; membranous: SSTR2), different analytic approaches have been chosen: The expression of Ki-67 and pHH3 was quantified as published [49] by using a standardized protocol of an image analysis system (IMS client, Imagic Bildverarbeitung AG, Glattbrugg, Switzerland). The expression patterns of HDAC5, chromogranin, serotonin, and insulin were analyzed with a semi-quantitative immunohistochemistry scoring system calculated by multiplication of the intensity and extensity yielding scores between 0 and 300 [50,51]. Finally, the expression analysis of SSTR2 was performed on the basis of an already published and routinely used scoring system ranging from 0 to 3 (0 = negative, 1 = cytoplasmatic, 2 = membranous incomplete, 3 = membranous circumferential; [52]).

### 4.6. Statistics

All data points represent mean values of at least three individual biological replicates ± standard error of mean (SEM) or Gaussian error propagation where applicable. Statistical outliers were excluded after identification using the Grubbs-test. ANOVA with a post-hoc Bonferroni analysis was used for calculation of significant differences between groups of samples with SPSS (IBM). Statistical results were considered significant (*) or highly significant (**) at *p* < 0.05 and *p* < 0.01, respectively. Data visualization were done with OriginPro 2017 (OriginLab, Northampton, MA, USA) and Corel Designer 2018 version 20.1.0.707 (Corel, Munich, Germany).
